# Supplementary material for: The origin of the central dogma through conflicting multilevel selection
Source: Proc Biol Sci. 2019 Oct 2;286(1912):20191359. doi: 10.1098/rspb.2019.1359 (PMC6790754; doi:10.1098/rspb.2019.1359)
Supplement: Supplementary Texts and Figures [file rspb20191359supp1.pdf]

Supplementary Material to "The origin of the central dogma through conflicting multilevel selection" in Proceedings of the Royal Society B http://dx.doi.org/10.1098/rspb.2019.1359

Nobuto Takeuchi*[1,2] and Kunihiko Kaneko[1,3]

[1]Research Center for Complex Systems Biology, Graduate School of Arts and Sciences, University of Tokyo, Komaba 3-8-1, Meguro-ku, Tokyo 153-8902, Japan
[2]School of Biological Sciences, Faculty of Science, University of Auckland, Private Bag 92019, Auckland 1142, New Zealand
[3]Department of Basic Science, Graduate School of Arts and Sciences, University of Tokyo, Komaba 3-8-1, Meguro-ku, Tokyo 153-8902, Japan

1 Supporting Texts

1.1 An alternative agent-based model in which coexistence between P and Q is selectively neutral

In this section, we describe an alternative agent-based model in which coexistence between P and Q is neutral with respect to cellular-level selection. In the agent-based model described in the main text, coexistence between P and Q is favoured by cellular-level selection. This is due to a specific rule about complex formation, which implies that replicators multiply fastest if both P and Q provide and receive catalysis (see Methods for details). To ascertain that this specific rule about complex formation does not critically affect results, we additionally examined an alternative model in which replicators multiply fastest even if only either P or Q provides and receives catalysis. In this model, cellular-level selection does not favour coexistence between P and Q while it still tends to maximise the multiplication rate of replicators within protocells.

In the alternative model, the reaction rate constants of complex formation are defined as a function of the k^c_{pt} values of a replicator serving as a catalyst as follows:

$$\max(k^c_{\mathrm{P}t}, k^c_{\mathrm{Q}t})\frac{k^c_{pt}}{k^c_{\mathrm{P}t} + k^c_{\mathrm{Q}t}}.$$

*Corresponding author. E-mail: nobuto.takeuchi@auckland.ac.nz

Under this definition, two replicators, denoted by X and Y, form a complex at a rate proportional to $\max(k^x_{\mathrm{P}y}, k^x_{\mathrm{Q}y}) + \max(k^y_{\mathrm{P}x}, k^y_{\mathrm{Q}x}) \leqslant 2k_{\max}$ if all possible complexes are considered, where x and y are the replicator types of X and Y, respectively (in the original model, this rate is proportional to $\sum_p k^x_{py} + k^y_{px} \leqslant 4k_{\max}$). Accordingly, replicators multiply fastest not only if $k^c_{pt} = k_{\max}$ for all combinations of c, p, and t, but also if $k^c_{cc} = k_{\max}$ for either $c = \mathrm{P}$ or $c = \mathrm{Q}$ and $k^c_{pt} = 0$ for all the other combinations of c, p, and t. In other words, replicators multiply fastest even if only either P or Q provides and receives catalysis (this is in contrast to the model described in the main text). While cellular-level selection always tends to maximise the multiplication rate of replicators within protocells, it is indifferent to how this maximisation is achieved. Therefore, cellular-level selection does not necessarily tend to maximise k^c_{pt} values for all combinations of c, p, and t; i.e., it does not necessarily favour coexistence between P and Q.

To examine the effect of coexistence between P and Q on symmetry breaking, we simulated the alternative model described above with two initial conditions, symmetric and asymmetric. In the symmetric initial condition, both P and Q were present—this is the same initial condition as used for the original agent-based model. In the asymmetric initial condition, only Q was present (see Fig. S2 for details)—this condition might be closer to what is typically imagined in the RNA world hypothesis. For both initial conditions, the model displays the same three-fold symmetry breaking as displayed by the original model (Fig. S2), indicating that the results do not depend on whether coexistence between P and Q is favoured by cellular-level selection.

1.2 Alternative agent-based models in which the mutation of k^c_{pt} is modelled differently

In this section, we describe alternative models for the mutation of k^c_{pt}. In the agent-based model described in the main text, the mutation of k^c_{pt} is modelled as unbiased random walks in a half-open interval $(-\infty, k_{\max})$ with a reflecting boundary at $k^c_{pt} = k_{\max}$. To ascertain that this specific model of mutation does not critically affect results, we additionally examined two alternative models of mutation. The first alternative model is nearly the same as the above, except that the reflecting boundary condition is set at $k^c_{pt} = 0$. In the second alternative model, each k^c_{pt} value is mutated by multiplying $\exp(\epsilon)$, where ϵ is a number randomly drawn from a uniform distribution on the interval $(-\delta_{\mathrm{mut}}, \delta_{\mathrm{mut}})$, with a reflecting boundary at $k^c_{pt} = k_{\max}$. Both models of mutation produce essentially the same result as described in the main text (Figs. S3 and S4), indicating that the results do not depend on the specific models of mutation.

1.3 The derivation of equation (1)

In this section, we describe the derivation of equations (1) that is outlined in Methods.

To derive equations (1), we simplified the agent-based model in two ways. First, we assumed that k^c_{pt} is independent of p and t. Under this assumption, a catalyst does not distinguish the replicator types of templates (i.e., $k^c_{pt} = k^c_{pt'}$ for $t \neq t'$) and products (i.e., $k^c_{pt} = k^c_{p't}$ for $p \neq p'$). This assumption excludes the possibility of numerical symmetry breaking, but still allows catalytic and informatic symmetry breaking as described in the main text (see Results).

Second, we abstracted away chemical reactions by defining ω^t_{ij} as the probability that replicator j of type t in protocell i is replicated or transcribed per unit time. Let $n^t_{ij}(\tau)$

be the population size of this replicator at time τ. Then, the dynamics of $n^t_{ij}(\tau)$ can be mathematically described as

$$\begin{bmatrix} n^{\mathrm{P}}_{ij}(\tau+1) \\ n^{\mathrm{Q}}_{ij}(\tau+1) \end{bmatrix} = \begin{bmatrix} \omega^{\mathrm{P}}_{ij} & \omega^{\mathrm{Q}}_{ij} \\ \omega^{\mathrm{P}}_{ij} & \omega^{\mathrm{Q}}_{ij} \end{bmatrix} \begin{bmatrix} n^{\mathrm{P}}_{ij}(\tau) \\ n^{\mathrm{Q}}_{ij}(\tau) \end{bmatrix}. \tag{S1}$$

The fitness of the replicator can be defined as the dominant eigenvalue λ_{ij} of the 2×2 matrix on the right-hand side of equation (S1). The equilibrium frequencies of P and Q are given by the right eigenvector $\boldsymbol{v}_{ij}$ associated with λ_{ij}. Fisher's reproductive values of P and Q are given by the corresponding left eigenvector $\boldsymbol{u}_{ij}$. These eigenvalue and eigenvectors are calculated as follows:

$$\lambda_{ij} = \omega^{\mathrm{P}}_{ij} + \omega^{\mathrm{Q}}_{ij}, \qquad \boldsymbol{v}_{ij} = \begin{bmatrix} 1 \\ 1 \end{bmatrix}, \qquad \boldsymbol{u}_{ij} = \begin{bmatrix} \omega^{\mathrm{P}}_{ij} & \omega^{\mathrm{Q}}_{ij} \end{bmatrix}. \tag{S2}$$

Based on the above simplification, we now derive equations (1). For concreteness, we focus on the evolution of the average catalytic activity of P (denoted by $\bar{k}^{\mathrm{P}}$ in the main text). However, the same method of derivation is applicable to that of Q if P and Q are swapped.

Let κ^{P}_{ij} be the catalytic activity of replicator j of type P in protocell i (we use κ instead of k to distinguish κ^{P}_{ij} from k^{P}_{pt}). Price's equation [1, 2] states that

$$\langle \lambda_{\tilde{i}\tilde{j}} \rangle \Delta \langle \kappa^{\mathrm{P}}_{\tilde{i}\tilde{j}} \rangle = \sigma^2_{\tilde{i}}[\langle \lambda_{i\tilde{j}} \rangle, \langle \kappa^{\mathrm{P}}_{i\tilde{j}} \rangle] + \mathbb{E}_{\tilde{i}}\big[\sigma^2_{\tilde{i}\tilde{j}}[\lambda_{ij}, \kappa^{\mathrm{P}}_{ij}]\big] \tag{S3}$$

where $\langle x_{i\tilde{j}} \rangle$, $\langle x_{\tilde{i}\tilde{j}} \rangle$, and $\mathbb{E}_{\tilde{i}}[x]$ are x averaged over the indices marked with tildes, $\sigma^2_{\tilde{i}}[x, y]$ is the covariance between x and y over protocells, and $\sigma^2_{i\tilde{j}}[x, y]$ is the covariance between x and y over the replicators in protocell i (one replicator is always counted as one sample in calculating all moments). Below, we show that equation (S3) is approximated by equations (1) up to the second moments of $\langle \kappa^{\mathrm{P}}_{i\tilde{j}} \rangle$ and κ^{P}_{ij}, namely, $\sigma^2_{\tilde{i}}[\langle \kappa^{\mathrm{P}}_{i\tilde{j}} \rangle, \langle \kappa^{\mathrm{P}}_{i\tilde{j}} \rangle]$ and $\mathbb{E}_{\tilde{i}}[\sigma^2_{i\tilde{j}}[\kappa^{\mathrm{P}}_{ij}, \kappa^{\mathrm{P}}_{ij}]]$.

To approximate the first term on the right-hand side of equation (S3), we assume that $\langle \lambda_{i\tilde{j}} \rangle$ is a function of $\langle \kappa^{\mathrm{P}}_{i\tilde{j}} \rangle$ and $\langle \kappa^{\mathrm{Q}}_{i\tilde{j}} \rangle$ that can be expanded as a Taylor series around $\langle \kappa^{\mathrm{P}}_{\tilde{i}\tilde{j}} \rangle$ and $\langle \kappa^{\mathrm{Q}}_{\tilde{i}\tilde{j}} \rangle$. Substituting this series into $\sigma^2_{\tilde{i}}[\langle \lambda_{i\tilde{j}} \rangle, \langle \kappa^{\mathrm{P}}_{i\tilde{j}} \rangle]$, we obtain

$$\sigma^2_{\tilde{i}}[\langle \lambda_{i\tilde{j}} \rangle, \langle \kappa^{\mathrm{P}}_{i\tilde{j}} \rangle] = \sum_{c \in \{\mathrm{P},\mathrm{Q}\}} \frac{\partial \langle \lambda_{\tilde{i}\tilde{j}} \rangle}{\partial \langle \kappa^{c}_{\tilde{i}\tilde{j}} \rangle} \sigma^2_{\tilde{i}}[\langle \kappa^{\mathrm{P}}_{i\tilde{j}} \rangle, \langle \kappa^{c}_{i\tilde{j}} \rangle] + O(\sigma^3_{\tilde{i}}), \tag{S4}$$

where $O(\sigma^3_{\tilde{i}})$ consists of terms involving the third or higher (mixed) central moments of $\langle \kappa^{\mathrm{P}}_{i\tilde{j}} \rangle$ and $\langle \kappa^{\mathrm{Q}}_{i\tilde{j}} \rangle$ over protocells [3].

To approximate the second term on the right-hand side of equation (S3), we likewise assume that λ_{ij} is a function of κ^{P}_{ij} and κ^{Q}_{ij} that can be expanded as a Taylor series around $\langle \kappa^{\mathrm{P}}_{i\tilde{j}} \rangle$ and $\langle \kappa^{\mathrm{Q}}_{i\tilde{j}} \rangle$. Substituting this series into $\sigma^2_{i\tilde{j}}[\lambda_{ij}, \kappa^{\mathrm{P}}_{ij}]$, we obtain

$$\sigma^2_{i\tilde{j}}[\lambda_{ij}, \kappa^{\mathrm{P}}_{ij}] = \sum_{c \in \{\mathrm{P},\mathrm{Q}\}} \frac{\partial \lambda_{ij}}{\partial \kappa^{c}_{ij}} \sigma^2_{i\tilde{j}}[\kappa^{\mathrm{P}}_{ij}, \kappa^{c}_{ij}] + O(\sigma^3_{i\tilde{j}}),$$

where $O(\sigma^3_{i\tilde{j}})$ consists of terms involving the third or higher (mixed) central moments of κ^{P}_{ij} and κ^{Q}_{ij} over the replicators in protocell i [3]. Applying $\mathbb{E}_{\tilde{i}}$ to both sides of the above

equation and assuming that $\partial\lambda_{ij}/\partial\kappa_{ij}^{c}$ is independent of $\sigma_{\tilde{i}\tilde{j}}^{2}[\kappa_{ij}^{\mathrm{P}},\kappa_{ij}^{c}]$, we obtain

$$\mathbb{E}_{\tilde{i}}\big[\sigma_{\tilde{i}\tilde{j}}^{2}[\lambda_{ij},\kappa_{ij}^{\mathrm{P}}]\big]=\sum_{c\in\{\mathrm{P},\mathrm{Q}\}}\mathbb{E}_{\tilde{i}}\left[\frac{\partial\lambda_{ij}}{\partial\kappa_{ij}^{c}}\right]\mathbb{E}_{\tilde{i}}\big[\sigma_{\tilde{i}\tilde{j}}^{2}[\kappa_{ij}^{\mathrm{P}},\kappa_{ij}^{c}]\big]+\mathbb{E}_{\tilde{i}}\big[O(\sigma_{\tilde{i}\tilde{j}}^{3})\big]. \tag{S5}$$

Substituting equations (S4) and (S5) into equation (S3), we obtain

$$\Delta\langle\kappa_{\tilde{i}\tilde{j}}^{\mathrm{P}}\rangle=\frac{1}{\langle\lambda_{\tilde{i}\tilde{j}}\rangle}\sum_{c\in\{\mathrm{P},\mathrm{Q}\}}\left(\frac{\partial\langle\lambda_{\tilde{i}\tilde{j}}\rangle}{\partial\langle\kappa_{\tilde{i}\tilde{j}}^{c}\rangle}\sigma_{\tilde{i}}^{2}[\langle\kappa_{\tilde{i}\tilde{j}}^{\mathrm{P}}\rangle,\langle\kappa_{\tilde{i}\tilde{j}}^{c}\rangle]+\mathbb{E}_{\tilde{i}}\left[\frac{\partial\lambda_{ij}}{\partial\kappa_{ij}^{c}}\right]\mathbb{E}_{\tilde{i}}\big[\sigma_{\tilde{i}\tilde{j}}^{2}[\kappa_{ij}^{\mathrm{P}},\kappa_{ij}^{c}]\big]\right)+O', \tag{S6}$$

where $O'=O(\sigma_{\tilde{i}}^{3})+E_{\tilde{i}}[O(\sigma_{\tilde{i}\tilde{j}}^{3})]$.

Next, we assume that covariances $\sigma_{\tilde{i}}^{2}[\langle\kappa_{\tilde{i}\tilde{j}}^{\mathrm{P}}\rangle,\langle\kappa_{\tilde{i}\tilde{j}}^{\mathrm{Q}}\rangle]$ and $\mathbb{E}_{\tilde{i}}\big[\sigma_{\tilde{i}\tilde{j}}^{2}[\kappa_{ij}^{\mathrm{P}},\kappa_{ij}^{\mathrm{Q}}]\big]$ are negligible because the mutation of κ_{ij}^{P} and that of κ_{ij}^{Q} are uncorrelated in the simulation model (this assumption is alternatively justified in Supplementary Material Text 1.6). Under this assumption, equation (S6) is transformed into

$$\Delta\langle\kappa_{\tilde{i}\tilde{j}}^{\mathrm{P}}\rangle=\frac{1}{\langle\lambda_{\tilde{i}\tilde{j}}\rangle}\left(\frac{\partial\langle\lambda_{\tilde{i}\tilde{j}}\rangle}{\partial\langle\kappa_{\tilde{i}\tilde{j}}^{\mathrm{P}}\rangle}\sigma_{\tilde{i}}^{2}[\langle\kappa_{\tilde{i}\tilde{j}}^{\mathrm{P}}\rangle,\langle\kappa_{\tilde{i}\tilde{j}}^{\mathrm{P}}\rangle]+\mathbb{E}_{\tilde{i}}\left[\frac{\partial\lambda_{ij}}{\partial\kappa_{ij}^{\mathrm{P}}}\right]\mathbb{E}_{\tilde{i}}\big[\sigma_{\tilde{i}\tilde{j}}^{2}[\kappa_{ij}^{\mathrm{P}},\kappa_{ij}^{\mathrm{P}}]\big]\right)+O'. \tag{S7}$$

Using equation (S2) (i.e., $\lambda_{ij}=\omega_{ij}^{\mathrm{P}}+\omega_{ij}^{\mathrm{Q}}$), we can transform equation (S7) into

$$\Delta\langle\kappa_{\tilde{i}\tilde{j}}^{\mathrm{P}}\rangle=\frac{1}{\langle\lambda_{\tilde{i}\tilde{j}}\rangle}\sum_{t\in\{\mathrm{P},\mathrm{Q}\}}\left(\frac{\partial\langle\omega_{\tilde{i}\tilde{j}}^{t}\rangle}{\partial\langle\kappa_{\tilde{i}\tilde{j}}^{\mathrm{P}}\rangle}\sigma_{\tilde{i}}^{2}[\langle\kappa_{\tilde{i}\tilde{j}}^{\mathrm{P}}\rangle,\langle\kappa_{\tilde{i}\tilde{j}}^{\mathrm{P}}\rangle]+\mathbb{E}_{\tilde{i}}\left[\frac{\partial\omega_{ij}^{t}}{\partial\kappa_{ij}^{\mathrm{P}}}\right]\mathbb{E}_{\tilde{i}}\big[\sigma_{\tilde{i}\tilde{j}}^{2}[\kappa_{ij}^{\mathrm{P}},\kappa_{ij}^{\mathrm{P}}]\big]\right)+O'. \tag{S8}$$

Moreover, it can be shown that

$$\begin{aligned}\mathbb{E}_{\tilde{i}}\left[\frac{\partial\omega_{ij}^{t}}{\partial\kappa_{ij}^{c}}\bigg|_{\substack{\kappa_{ij}^{\mathrm{P}}=\langle\kappa_{\tilde{i}\tilde{j}}^{\mathrm{P}}\rangle\\ \kappa_{ij}^{\mathrm{Q}}=\langle\kappa_{\tilde{i}\tilde{j}}^{\mathrm{Q}}\rangle}}\right]&=\mathbb{E}_{\tilde{i}}\left[\omega_{ij}^{t}(\langle\kappa_{\tilde{i}\tilde{j}}^{\mathrm{P}}\rangle,\langle\kappa_{\tilde{i}\tilde{j}}^{\mathrm{Q}}\rangle)\frac{\partial\ln\omega_{ij}^{t}}{\partial\kappa_{ij}^{c}}\bigg|_{\substack{\kappa_{ij}^{\mathrm{P}}=\langle\kappa_{\tilde{i}\tilde{j}}^{\mathrm{P}}\rangle\\ \kappa_{ij}^{\mathrm{Q}}=\langle\kappa_{\tilde{i}\tilde{j}}^{\mathrm{Q}}\rangle}}\right]\\&=\mathbb{E}_{\tilde{i}}\big[\omega_{ij}^{t}(\langle\kappa_{\tilde{i}\tilde{j}}^{\mathrm{P}}\rangle,\langle\kappa_{\tilde{i}\tilde{j}}^{\mathrm{Q}}\rangle)\big]\mathbb{E}_{\tilde{i}}\left[\frac{\partial\ln\omega_{ij}^{t}}{\partial\kappa_{ij}^{c}}\bigg|_{\substack{\kappa_{ij}^{\mathrm{P}}=\langle\kappa_{\tilde{i}\tilde{j}}^{\mathrm{P}}\rangle\\ \kappa_{ij}^{\mathrm{Q}}=\langle\kappa_{\tilde{i}\tilde{j}}^{\mathrm{Q}}\rangle}}\right]+O(\sigma_{\tilde{i}}^{2})\\&=\langle\omega_{\tilde{i}\tilde{j}}^{t}\rangle\mathbb{E}_{\tilde{i}}\left[\frac{\partial\ln\omega_{ij}^{t}}{\partial\kappa_{ij}^{c}}\bigg|_{\substack{\kappa_{ij}^{\mathrm{P}}=\langle\kappa_{\tilde{i}\tilde{j}}^{\mathrm{P}}\rangle\\ \kappa_{ij}^{\mathrm{Q}}=\langle\kappa_{\tilde{i}\tilde{j}}^{\mathrm{Q}}\rangle}}\right]+\mathbb{E}_{\tilde{i}}\big[O(\sigma_{\tilde{i}\tilde{j}}^{2})\big]+O(\sigma_{\tilde{i}}^{2}).\end{aligned}$$

Using the above equation, we can transform equation (S8) into

$$\Delta\langle\kappa_{\tilde{i}\tilde{j}}^{\mathrm{P}}\rangle=\sum_{t\in\{\mathrm{P},\mathrm{Q}\}}\frac{\langle\omega_{\tilde{i}\tilde{j}}^{t}\rangle}{\langle\lambda_{\tilde{i}\tilde{j}}\rangle}\left(\frac{\partial\ln\langle\omega_{\tilde{i}\tilde{j}}^{t}\rangle}{\partial\langle\kappa_{\tilde{i}\tilde{j}}^{\mathrm{P}}\rangle}\sigma_{\tilde{i}}^{2}[\langle\kappa_{\tilde{i}\tilde{j}}^{\mathrm{P}}\rangle,\langle\kappa_{\tilde{i}\tilde{j}}^{\mathrm{P}}\rangle]+\mathbb{E}_{\tilde{i}}\left[\frac{\partial\ln\omega_{ij}^{t}}{\partial\kappa_{ij}^{\mathrm{P}}}\right]\mathbb{E}_{\tilde{i}}\big[\sigma_{\tilde{i}\tilde{j}}^{2}[\kappa_{ij}^{\mathrm{P}},\kappa_{ij}^{\mathrm{P}}]\big]\right)+O'', \tag{S9}$$

where $O''=O'+O(\sigma_{\tilde{i}}^{2})\ \mathbb{E}_{\tilde{i}}[O(\sigma_{\tilde{i}\tilde{j}}^{2})]+\mathbb{E}_{\tilde{i}}[O(\sigma_{\tilde{i}\tilde{j}}^{2})]\mathbb{E}_{\tilde{i}}[O(\sigma_{\tilde{i}\tilde{j}}^{2})]$.

We adopt the following notation:

$$\bar{\omega}^{t}=\frac{\langle\omega_{\tilde{i}\tilde{j}}^{t}\rangle}{\langle\lambda_{\tilde{i}\tilde{j}}\rangle},\qquad\sigma_{\mathrm{cel}}^{2}=\sigma_{\tilde{i}}^{2}[\langle\kappa_{\tilde{i}\tilde{j}}^{\mathrm{P}}\rangle,\langle\kappa_{\tilde{i}\tilde{j}}^{\mathrm{P}}\rangle],\qquad\sigma_{\mathrm{mol}}^{2}=\mathbb{E}_{\tilde{i}}[\sigma_{\tilde{i}\tilde{j}}^{2}[\kappa_{ij}^{\mathrm{P}},\kappa_{ij}^{\mathrm{P}}]],$$

$$\bar{k}^{\mathrm{P}}=\langle\kappa_{\tilde{i}\tilde{j}}^{\mathrm{P}}\rangle,\qquad\gamma_{\mathrm{P}}^{\mathrm{P}}=-\mathbb{E}_{\tilde{i}}\left[\frac{\partial\ln\omega_{ij}^{\mathrm{P}}}{\partial\kappa_{ij}^{\mathrm{P}}}\right],\qquad\beta_{\mathrm{P}}^{t}=\frac{\partial\ln\langle\omega_{\tilde{i}\tilde{j}}^{t}\rangle}{\partial\langle\kappa_{\tilde{i}\tilde{j}}^{\mathrm{P}}\rangle},$$

where $\bar{\omega}^t$ is the normalised average reproductive value of type-t replicators, $\sigma^2_{\rm cel}$, $\sigma^2_{\rm mol}$, and $\bar{k}^{\rm P}$ are the simplification of the notation, $\gamma^{\rm P}_{\rm P}$ is an average decrease in the replication rate of a type-P replicator due to an increase in its own catalytic activity, and $\beta^t_{\rm P}$ is an increase in the average replication rate of type-t replicators in a protocell due to an increase in the average catalytic activity of type-P replicators in that protocell.

We assume that V is so large that $\langle\kappa^{\rm P}_{i\tilde{j}}\rangle$ and $\kappa^{\rm P}_{ij}$ can be regarded as mathematically independent of each other, provided i and j are fixed (if i and j are varied, $\langle\kappa^{\rm P}_{i\tilde{j}}\rangle$ and $\kappa^{\rm P}_{ij}$ may be statistically correlated). Under this assumption, increasing $\kappa^{\rm P}_{ij}$ does not increase $\langle\kappa^{\rm P}_{i\tilde{j}}\rangle$, so that $\gamma^{\rm P}_{\rm P}$ reflects only the cost of providing catalysis at the molecular level. Likewise, increasing $\langle\kappa^{\rm P}_{i\tilde{j}}\rangle$ does not increase $\kappa^{\rm P}_{ij}$, so that $\beta^t_{\rm P}$ reflects only the benefit of receiving catalysis at the cellular level. Moreover, the independence of $\langle\kappa^{\rm P}_{i\tilde{j}}\rangle$ from $\kappa^{\rm P}_{ij}$ implies that $\partial\omega^{\rm Q}_{ij}/\partial\kappa^{\rm P}_{ij} = 0$, which permits the following interpretation: if a replicator of type P provides more catalysis, its transcripts, which is of type Q, pay no extra cost (i.e., $\gamma^{\rm Q}_{\rm P} = 0$).

Using the above notation and the fact that $\partial\omega^{\rm Q}_{ij}/\partial\kappa^{\rm P}_{ij} = 0$, we can transform equation (S9) into

$$\Delta\bar{k}^{\rm P} \approx \bar{\omega}^{\rm P}(b^{\rm P}_{\rm P}\sigma^2_{\rm cel} - \gamma^{\rm P}_{\rm P}\sigma^2_{\rm mol}) + \bar{\omega}^{\rm Q}b^{\rm Q}_{\rm P}\sigma^2_{\rm cel}, \tag{S10}$$

where O'' is omitted. Equation (S10) is identical to equations (1).

Finally, to derive the equation for $\Delta\bar{k}^{\rm Q}$ (i.e., $\Delta\langle\kappa^{\rm Q}_{\tilde{i}\tilde{j}}\rangle$), we swap P and Q in the above derivation. Moreover, we assume that $\sigma^2_{\tilde{i}}[\langle\kappa^{\rm Q}_{i\tilde{j}}\rangle, \langle\kappa^{\rm Q}_{i\tilde{j}}\rangle] = \sigma^2_{\tilde{i}}[\langle\kappa^{\rm P}_{i\tilde{j}}\rangle, \langle\kappa^{\rm P}_{i\tilde{j}}\rangle]$ and $\mathbb{E}_{\tilde{i}}[\sigma^2_{i\tilde{j}}[\kappa^{\rm Q}_{ij}, \kappa^{\rm Q}_{ij}]] = \mathbb{E}_{\tilde{i}}[\sigma^2_{i\tilde{j}}[\kappa^{\rm P}_{ij}, \kappa^{\rm P}_{ij}]]$ because no difference is a priori assumed between P and Q.

1.4 The mathematical analysis of numerical symmetry breaking

In this section, we show that numerical symmetry breaking occurs because while it is neither favoured nor disfavoured by molecular-level selection, it is favoured by cellular-level selection if catalytic and informatic symmetry breaking has occurred. To this end, we will again simplify the agent-based model into mathematical equations in a mannar analogous to that used to derive equations (1).

Before describing the mathematical analysis, we first need to note that the proximate—as opposed to ultimate—cause of numerical symmetry breaking is the self-replication of catalysts (i.e., $k^c_{cc} > 0$, where c is the replicator type of catalysts) in the absence of the reverse transcription of catalysts (i.e., $k^c_{tc} = 0$, where t is the replicator type of templates). This fact can be inferred from the following two results. First, when catalytic, informatic, and numerical symmetry breaking occurs, the replication and transcription of templates are catalysed at about the same rate, i.e., $k^c_{tt} \approx k^c_{ct}$ (Fig. 2b). Therefore, the replication and transcription of templates cannot cause numerical asymmetry. Second, when catalytic and informatic symmetry breaking occurs without numerical symmetry breaking, the self-replication of catalysts is absent (Fig. S5). Taken together, these results indicate that the proximate cause of numerical symmetry breaking is the self-replication of catalysts in the absence of the reverse transcription of catalysts. Therefore, to understand why numerical symmetry breaking occurs, we need to understand why the self-replication of catalysts evolves if catalytic and informatic symmetry breaking has occurred.

To address the above question, we assume that replicators have already undergone catalytic and informatic symmetry breaking and consider how the fitness of those replicators depends on the self-replication of catalysts. The population dynamics of replicators

with catalytic and informatic asymmetry can be described as follows. Let $n^t_{ij}(\tau)$ be the population size of replicator j of type t in protocell i at time τ. Let catalysts and templates be P and Q, respectively. Then, the dynamics of $n^t_{ij}(\tau)$ is mathematically described as follows:

$$\begin{bmatrix} n^{\mathrm{P}}_{ij}(\tau+1) \\ n^{\mathrm{Q}}_{ij}(\tau+1) \end{bmatrix} = \begin{bmatrix} w^{\mathrm{PP}}_{ij} & \omega^{\mathrm{Q}}_{ij} \\ 0 & \omega^{\mathrm{Q}}_{ij} \end{bmatrix} \begin{bmatrix} n^{\mathrm{P}}_{ij}(\tau) \\ n^{\mathrm{Q}}_{ij}(\tau) \end{bmatrix}, \tag{S11}$$

where w^{PP}_{ij} is the self-replication probability of catalysts, and ω^{Q}_{ij} is the replication and transcription probabilities of templates, which are assumed to be identical to each other. The fitness of replicators can be defined as the dominant eigenvalue (denoted by λ_{ij}) of the 2×2 matrix on the right-hand side of equation (S11):

$$\lambda_{ij} = \begin{cases} \omega^{\mathrm{Q}}_{ij} & \text{if } \omega^{\mathrm{Q}}_{ij} > w^{\mathrm{PP}}_{ij} \\ w^{\mathrm{PP}}_{ij} & \text{otherwise.} \end{cases} \tag{S12}$$

The associated right eigenvector, which determines the stationary frequencies of P and Q, is

$$\boldsymbol{v}_{ij} = \begin{cases} \frac{1}{2 - w^{\mathrm{PP}}_{ij}/\omega^{\mathrm{Q}}_{ij}} \begin{bmatrix} 1 \\ 1 - w^{\mathrm{PP}}_{ij}/\omega^{\mathrm{Q}}_{ij} \end{bmatrix} & \text{if } \omega^{\mathrm{Q}}_{ij} > w^{\mathrm{PP}}_{ij} \\ \begin{bmatrix} 1 \\ 0 \end{bmatrix} & \text{otherwise.} \end{cases} \tag{S13}$$

Equation (S13) shows that we must assume $\omega^{\mathrm{Q}}_{ij} > w^{\mathrm{PP}}_{ij}$ in order for P and Q to coexist. Equation (S13) also shows that the frequency of catalysts (i.e., $1/(2 - w^{\mathrm{PP}}_{ij}/\omega^{\mathrm{Q}}_{ij})$) increases with the self-replication of catalysts (i.e., w^{PP}_{ij}), as stated in the beginning of this section.

We first examine whether the self-replication of catalysts is favoured by molecular-level selection. To this end, we consider how the fitness of replicators (i.e., λ_{ij}) depends on the self-replication of catalysts (i.e., w^{PP}_{ij}). According to equation (S12), λ_{ij} does not directly depend on w^{PP}_{ij}. However, λ_{ij} can indirectly depend on w^{PP}_{ij} because λ_{ij} increases with the frequency of catalysts in a protocell (i.e., $\mathbb{E}_{i\tilde{j}}[1/(2 - w^{\mathrm{PP}}_{i\tilde{j}}/\omega^{\mathrm{Q}}_{i\tilde{j}})]$). This frequency increases with w^{PP}_{ij} if V is so small that a particular replicator can influence the frequency of catalysts in the protocell. However, if λ_{ij} increases with w^{PP}_{ij}, the average fitness of replicators in the protocell (i.e., $\langle \lambda_{i\tilde{j}} \rangle$) must also increase. Therefore, we need to consider the relative fitness (i.e., $\lambda_{ij}/\langle \lambda_{i\tilde{j}} \rangle$). The relative fitness is independent of w^{PP}_{ij} because catalysis is equally shared among templates within a protocell. Therefore, the self-replication of catalysts is neither favoured not disfavoured by molecular-level selection.

We next examine whether the self-replication of catalysts is favoured by cellular-level selection. To this end, we consider how the fitness of a protocell depends on the average self-replication of catalysts in that protocell (i.e., $\langle w^{\mathrm{PP}}_{i\tilde{j}} \rangle$). The fitness of a protocell can be defined as the average fitness of the replicators in that protocell (i.e., $\langle \lambda_{i\tilde{j}} \rangle$). According to equation (S12), $\langle \lambda_{i\tilde{j}} \rangle$ does not directly depend $\langle w^{\mathrm{PP}}_{i\tilde{j}} \rangle$. However, $\langle \lambda_{i\tilde{j}} \rangle$ indirectly depends on $\langle w^{\mathrm{PP}}_{i\tilde{j}} \rangle$ because $\langle \lambda_{i\tilde{j}} \rangle$ increases with the frequency of catalysts in a protocell (i.e., $\mathbb{E}_{i\tilde{j}}[1/(2 - w^{\mathrm{PP}}_{i\tilde{j}}/\omega^{\mathrm{Q}}_{i\tilde{j}})]$). This frequency increases with $\langle w^{\mathrm{PP}}_{i\tilde{j}} \rangle$, so that $\langle \lambda_{i\tilde{j}} \rangle$ must also increase with $\langle w^{\mathrm{PP}}_{i\tilde{j}} \rangle$. Therefore, the self-replication of catalysts is favoured by cellular-level selection.

Taken together, the above considerations indicate that the self-replication of catalysts is neutral with respect to molecular-level selection, but advantageous with respect to

cellular-level selection. Therefore, numerical symmetry breaking results from the maximisation of fitness at the cellular level in the presence of catalytic and informatic asymmetry.

Finally, we mention an important consequence of numerical symmetry breaking. Numerical symmetry breaking causes a bottleneck effect on the population of replicators within a protocell. This bottleneck effect increases among-cell variance relative to within-cell variance (i.e., $\sigma^2_{\text{cel}}/\sigma^2_{\text{mol}}$); therefore, it has a stabilising effect on protocells [4, 5]. In this regard, numerical symmetry breaking can be compared to life-cycle bottlenecks displayed by multicellular organisms and eusocial colonies (i.e., an organism or colony develops from only one or a few propagules), which are considered to reduce within-group conflict [6–8].

1.5 The hierarchical Wright-Fisher model

In this section, we describe a model that stochastically simulates the population dynamics described by equations (1), in which σ^2_{mol} and σ^2_{cel} are treated as dynamic variables dependent on m and V.

The simplifications involved in the derivation of equations (1), while illuminating, make the comparison between equations (1) and the agent-based model indirect. Specifically, equations (1) cannot be compared with the agent-based model in terms of the same parameters, because the equations treat σ^2_{mol} and σ^2_{cel} as parameters, which are actually dynamic variables dependent on m and V in the agent-based model. To fill this gap, we constructed a model that stochastically simulates the population dynamics described by equations (1) and treats σ^2_{mol} and σ^2_{cel} as dynamic variables dependent on m and V.

This model is formulated as a hierarchical Wright-Fisher process. Replicators are partitioned into a number of groups (hereafter, protocells). Each replicator is individually assigned replicator type $c \in \{\text{P}, \text{Q}\}$ and two k^c values. The fitness of a replicator is calculated according to equation (S14). In each generation, replicators are replicated or transcribed with probabilities proportional to ω^c_{ij}, so that the population dynamics matches equation (S1) on average. After the replication-transcription step, the protocells containing greater than V replicators are divided with their replicators randomly distributed between the two daughter cells. The protocells containing no replicators are discarded.

The mutation of k^c is modelled as unbiased random walks with reflecting boundaries. That is, each k^c value of a replicator is mutated with a probability m per replication or transcription by adding a number randomly drawn from a uniform distribution on the interval $(-\delta_{\text{mut}}, \delta_{\text{mut}})$ ($\delta_{\text{mut}} = 0.1$). The values of k^c are bounded in $[0, 1]$ with reflecting boundaries at both bounds.

To determine the condition for symmetry breaking, we simulated the above Wright-Fisher model for various values of V and m. The simulations show that symmetry breaking occurs only if V and m are sufficiently large (Fig. S8), a result that is consistent with the outcomes of the original agent-based model (Fig. 2). Given that the Wright-Fisher model involves many of the simplifications involved in equations (1), the above consistency supports the validity of the symmetry breaking mechanism described by equations (1).

1.6 The phase-plane analysis

In this section, we describe the phase-plane analysis outlined in Methods.

To perform the phase-plane analysis depicted in Fig. 3, we adapted equations (1) by defining ω^t_{ij} as a specific function of κ^t_{ij} (see the previous section for the meaning of ω^t_{ij}

and κ^t_{ij}). The following definition was employed:

$$\omega^t_{ij} = e^{\langle\kappa^{\rm P}_{\tilde{i}\tilde{j}}\rangle + \langle\kappa^{\rm Q}_{\tilde{i}\tilde{j}}\rangle} \frac{e^{-s\kappa^t_{ij}}}{\langle e^{-s\kappa^{\rm P}_{\tilde{i}\tilde{j}}}\rangle + \langle e^{-s\kappa^{\rm Q}_{\tilde{i}\tilde{j}}}\rangle}. \tag{S14}$$

where the factor $e^{\langle\kappa^{\rm P}_{\tilde{i}\tilde{j}}\rangle + \langle\kappa^{\rm Q}_{\tilde{i}\tilde{j}}\rangle}$ represents the cellular-level benefit of catalysis provided by the replicators in protocell i, the numerator $e^{-s\kappa^t_{ij}}$ represents the molecular-level cost of catalysis provided by the focal replicator, the denominator $1/(\langle e^{-s\kappa^{\rm P}_{\tilde{i}\tilde{j}}}\rangle + \langle e^{-s\kappa^{\rm Q}_{\tilde{i}\tilde{j}}}\rangle)$ normalises the cost, and s is the cost-benefit ratio. The above definition of ω^t_{ij} was chosen to satisfy the requirement that a replicator faces the trade-off between providing catalysis and serving as a template, so that γ^t_t and β^t_c are positive; for example, if the cost γ^t_t were negative, it would actually be a benefit, so that there would be no trade-off. This requirement is satisfied if $\partial\omega^t_{ij}/\partial\kappa^t_{ij} < 0$ and $\partial\langle\omega^t_{\tilde{i}\tilde{j}}\rangle/\partial\langle\kappa^c_{\tilde{i}\tilde{j}}\rangle > 0$ for $c = t$ and $c \neq t$. Apart from this requirement, the definition was arbitrarily chosen for simplicity.

Under the definition of ω^t_{ij} in equation (S14), we obtain equations describing the evolution of $\langle\kappa^c_{\tilde{i}\tilde{j}}\rangle$ (denoted as $\bar{k}^c$ in the main text) as follows. Since the evolution of $\langle\kappa^c_{\tilde{i}\tilde{j}}\rangle$ is described by equation (S6), we substitute equation (S14) into equation (S6). For this substitution, we need to calculate the derivatives of fitness. According to equation (S2), the fitness of a replicator is $\lambda_{ij} = \omega^{\rm P}_{ij} + \omega^{\rm Q}_{ij}$. Therefore,

$$\begin{aligned} \mathbb{E}_{\tilde{i}}\left[\frac{\partial\lambda_{ij}}{\partial\kappa^c_{ij}}\bigg|_{\substack{\kappa^{\rm P}_{ij}=\langle\kappa^{\rm P}_{\tilde{i}\tilde{j}}\rangle \\ \kappa^{\rm Q}_{ij}=\langle\kappa^{\rm Q}_{\tilde{i}\tilde{j}}\rangle}}\right] &= \mathbb{E}_{\tilde{i}}\left[-ce^{\langle\kappa^{\rm P}_{\tilde{i}\tilde{j}}\rangle + \langle\kappa^{\rm Q}_{\tilde{i}\tilde{j}}\rangle} \frac{e^{-s\langle\kappa^c_{\tilde{i}\tilde{j}}\rangle}}{\langle e^{-s\kappa^{\rm P}_{\tilde{i}\tilde{j}}}\rangle + \langle e^{-s\kappa^{\rm Q}_{\tilde{i}\tilde{j}}}\rangle}\right] \\ &= -ce^{\langle\kappa^{\rm P}_{\tilde{i}\tilde{j}}\rangle + \langle\kappa^{\rm Q}_{\tilde{i}\tilde{j}}\rangle} \frac{e^{-s\langle\kappa^c_{\tilde{i}\tilde{j}}\rangle}}{e^{-s\langle\kappa^{\rm P}_{\tilde{i}\tilde{j}}\rangle} + e^{-s\langle\kappa^{\rm Q}_{\tilde{i}\tilde{j}}\rangle}} + \mathbb{E}_{\tilde{i}}\big[O(\sigma^2_{\tilde{i}\tilde{j}})\big] + O(\sigma^2_{\tilde{i}}). \end{aligned}$$

Moreover, the average fitness of replicators in a protocell is $\langle\lambda_{i\tilde{j}}\rangle = e^{\langle\kappa^{\rm P}_{i\tilde{j}}\rangle + \langle\kappa^{\rm Q}_{i\tilde{j}}\rangle}$, so

$$\frac{\partial\langle\lambda_{i\tilde{j}}\rangle}{\partial\langle\kappa^c_{i\tilde{j}}\rangle}\bigg|_{\substack{\langle\kappa^{\rm P}_{i\tilde{j}}\rangle=\langle\kappa^{\rm P}_{\tilde{i}\tilde{j}}\rangle \\ \langle\kappa^{\rm Q}_{i\tilde{j}}\rangle=\langle\kappa^{\rm Q}_{\tilde{i}\tilde{j}}\rangle}} = e^{\langle\kappa^{\rm P}_{\tilde{i}\tilde{j}}\rangle + \langle\kappa^{\rm Q}_{\tilde{i}\tilde{j}}\rangle}.$$

We substitute these derivatives into equation (S6) and use the fact that

$$\langle\lambda_{\tilde{i}\tilde{j}}\rangle = e^{\langle\kappa^{\rm P}_{\tilde{i}\tilde{j}}\rangle + \langle\kappa^{\rm Q}_{\tilde{i}\tilde{j}}\rangle} + O(\sigma^2_{\tilde{i}})$$

to obtain

$$\Delta\langle\kappa^c_{\tilde{i}\tilde{j}}\rangle = (1+\rho_{\rm cel})\sigma^2_{\rm cel} - s\frac{e^{-s\langle\kappa^c_{\tilde{i}\tilde{j}}\rangle} + \rho_{\rm mol}e^{-s\langle\kappa^{c'}_{\tilde{i}\tilde{j}}\rangle}}{e^{-s\langle\kappa^{\rm P}_{\tilde{i}\tilde{j}}\rangle} + e^{-s\langle\kappa^{\rm Q}_{\tilde{i}\tilde{j}}\rangle}}\sigma^2_{\rm mol} + O'', \tag{S15}$$

where $c' \neq c$, $\rho_{\rm cel}$ is the correlation coefficient between $\langle\kappa^{\rm P}_{i\tilde{j}}\rangle$ and $\langle\kappa^{\rm Q}_{i\tilde{j}}\rangle$ (i.e., $\rho_{\rm cel} = \sigma^2_{\tilde{i}}[\langle\kappa^{\rm P}_{i\tilde{j}}\rangle, \langle\kappa^{\rm Q}_{i\tilde{j}}\rangle]/\sigma^2_{\rm cel}$), and $\rho_{\rm mol}$ is the average correlation coefficient between $\kappa^{\rm P}_{ij}$ and $\kappa^{\rm Q}_{ij}$ (i.e., $\rho_{\rm mol} = \mathbb{E}_{\tilde{i}}[\sigma^2_{i\tilde{j}}[\kappa^{\rm P}_{ij}, \kappa^{\rm Q}_{ij}]]/\sigma^2_{\rm mol}$). To derive equation (S15), we have assumed that the variances of $\langle\kappa^c_{i\tilde{j}}\rangle$ and κ^c_{ij} are independent of c; i.e., $\sigma^2_{\rm cel} = \sigma^2_{\tilde{i}}[\langle\kappa^c_{i\tilde{j}}\rangle, \langle\kappa^c_{i\tilde{j}}\rangle]$ and $\sigma^2_{\rm mol} = \mathbb{E}_{\tilde{i}}[\sigma^2_{i\tilde{j}}[\kappa^c_{ij}, \kappa^c_{ij}]]$ for $c = {\rm P}$ and $c = {\rm Q}$.

Equation (S15) can be expressed in a compact form as follows:

$$\begin{bmatrix}\Delta\langle\kappa^{\rm P}_{\tilde{i}\tilde{j}}\rangle \\ \Delta\langle\kappa^{\rm Q}_{\tilde{i}\tilde{j}}\rangle\end{bmatrix} = \sigma^2_{\rm tot}\nabla\big[RB - (1-R)C\big] + O'',$$

where ∇ is a nabla operator (i.e., $\nabla = [\partial/\partial\langle\kappa^{\mathrm{P}}_{\tilde{i}\tilde{j}}\rangle, \partial/\partial\langle\kappa^{\mathrm{Q}}_{\tilde{i}\tilde{j}}\rangle]^{\mathrm{T}}$, where ^{T} denotes transpose), $\sigma^2_{\mathrm{tot}} = \sigma^2_{\mathrm{mol}} + \sigma^2_{\mathrm{cel}}$, $R = \sigma^2_{\mathrm{cel}}/(\sigma^2_{\mathrm{cel}} + \sigma^2_{\mathrm{mol}})$, $B = (1 + \rho_{\mathrm{cel}})(\kappa^{\mathrm{P}}_{\tilde{i}\tilde{j}} + \kappa^{\mathrm{Q}}_{\tilde{i}\tilde{j}})$, and $C = (\rho_{\mathrm{mol}} - 1)\ln(e^{-s\kappa^{\mathrm{P}}_{\tilde{i}\tilde{j}}} + e^{-s\kappa^{\mathrm{Q}}_{\tilde{i}\tilde{j}}}) + \rho_{\mathrm{mol}} s(\kappa^{\mathrm{P}}_{\tilde{i}\tilde{j}} + \kappa^{\mathrm{Q}}_{\tilde{i}\tilde{j}})$. R can be interpreted as the regression coefficient of $\langle\kappa^{c}_{\tilde{i}\tilde{j}}\rangle$ on κ^{c}_{ij} [9] and, therefore, the coefficient of genetic relatedness [10]. The potential function $RB - (1-R)C$ can then be interpreted as inclusive fitness.

Next, we set $\rho_{\mathrm{mol}} = 0$ and $\rho_{\mathrm{cel}} = 0$ in equations (S15) and let $\langle\kappa^{c}_{\tilde{i}\tilde{j}}\rangle$ be denoted by $\bar{k}^c$, obtaining

$$\begin{aligned}\Delta\bar{k}^c &= \sigma^2_{\mathrm{cel}} - s\frac{e^{-s\bar{k}^c}}{e^{-s\bar{k}^{\mathrm{P}}} + e^{-s\bar{k}^{\mathrm{Q}}}}\sigma^2_{\mathrm{mol}} + O'' \\ &= \frac{e^{-s\bar{k}^c}}{e^{-s\bar{k}^{\mathrm{P}}} + e^{-s\bar{k}^{\mathrm{Q}}}}(\sigma^2_{\mathrm{cel}} - s\sigma^2_{\mathrm{mol}}) + \frac{e^{-s\bar{k}^{c'}}}{e^{-s\bar{k}^{\mathrm{P}}} + e^{-s\bar{k}^{\mathrm{Q}}}}\sigma^2_{\mathrm{cel}} + O'',\end{aligned} \tag{S16}$$

where $c' \neq c$. Comparing equations (S16) and (S10), we infer that

$$\begin{aligned}\bar{\omega}^c &= \frac{e^{-s\bar{k}^c}}{e^{-s\bar{k}^{\mathrm{P}}} + e^{-s\bar{k}^{\mathrm{Q}}}}, \\ \gamma^c_c &= s, \\ \beta^t_c &= 1,\end{aligned}$$

which are identical to equations (5).

Next, we omit O'' in equation (S16) and replace Δ with time derivative $d/d\tau$, obtaining

$$\frac{d}{d\tau}\bar{k}^c = \sigma^2_{\mathrm{cel}} - s\frac{e^{-s\bar{k}^c}}{e^{-s\bar{k}^{\mathrm{P}}} + e^{-s\bar{k}^{\mathrm{Q}}}}\sigma^2_{\mathrm{mol}}. \tag{S17}$$

Finally, to allow for the restriction on the range of $\bar{k}^c$ (i.e., $\bar{k}^c \in [0, k_{\max}]$), we multiply the right-hand side of equation (S17) with a function, denoted by $\Theta(\bar{k}^c)$, that is 1 if $0 < \bar{k}^c < k_{\max}$ and 0 if $\bar{k}^c = 0$ or $\bar{k}^c = k_{\max}$. Multiplying $\Theta(\bar{k}^c)$ with the right-hand side of equation (S17), we obtain

$$\frac{d}{d\tau}\bar{k}^c = \Theta(\bar{k}^c)\left[\sigma^2_{\mathrm{cel}} - s\frac{e^{-s\bar{k}^c}}{e^{-s\bar{k}^{\mathrm{P}}} + e^{-s\bar{k}^{\mathrm{Q}}}}\sigma^2_{\mathrm{mol}}\right].$$

The above equation was numerically integrated for $s = 1$ to obtain the phase-plane portrait depicted in Fig. 3.

Equation (S15) allows for statistical correlations between κ^{P}_{ij} and κ^{Q}_{ij} at the molecular and cellular levels, i.e., ρ_{mol} and ρ_{cel}. Therefore, it can be used to examine the consequence of ignoring these correlations, which is one of the simplifications made in the derivation of equations (1) described in Supplementary Material Text 1.3. For this sake, we calculate the nullcline of $\Delta\langle\kappa^{c}_{\tilde{i}\tilde{j}}\rangle$. Setting $\Delta\langle\kappa^{c}_{\tilde{i}\tilde{j}}\rangle = 0$ in equation (S15) and omitting O'', we obtain

$$\langle\kappa^{c'}_{\tilde{i}\tilde{j}}\rangle \approx \langle\kappa^{c}_{\tilde{i}\tilde{j}}\rangle + s^{-1}\ln\frac{\rho_{\mathrm{mol}} s\sigma^2_{\mathrm{mol}} - (1+\rho_{\mathrm{cel}})\sigma^2_{\mathrm{cel}}}{(1+\rho_{\mathrm{cel}})\sigma^2_{\mathrm{cel}} - s\sigma^2_{\mathrm{mol}}}.$$

This equation shows that all parameters only appear in the intercept of the nullcline with the $\langle\kappa^{c'}_{\tilde{i}\tilde{j}}\rangle$-axis. Let us denote this intercept as $s^{-1}\ln I$. The way I qualitatively depends on σ^2_{cel} and $s\sigma^2_{\mathrm{mol}}$ is independent of ρ_{cel} because $-1 < \rho_{\mathrm{cel}} < 1$. Therefore, we

can assume that $\rho_{\text{cel}} = 0$ without loss of generality. Next, to see how ρ_{mol} influences I, we focus on the singularity of I by setting $(1 + \rho_{\text{cel}})\sigma_{\text{cel}}^2 = s\sigma_{\text{mol}}^2 + \epsilon$, where $\epsilon > 0$. Then, $I = (1 - \rho_{\text{mol}})s\sigma_{\text{mol}}^2/\epsilon - \rho_{\text{mol}}$. The way I qualitatively depends on $s\sigma_{\text{mol}}^2/\epsilon$ is independent of ρ_{mol} because $-1 < \rho_{\text{mol}} < 1$. Therefore, we can assume that $\rho_{\text{mol}} = 0$ without loss of generality. Taken together, these calculations show that ignoring correlations between κ_{ij}^{P} and κ_{ij}^{Q} does not qualitatively affect the results, supporting the validity of equations (1).

2 Supporting Figures

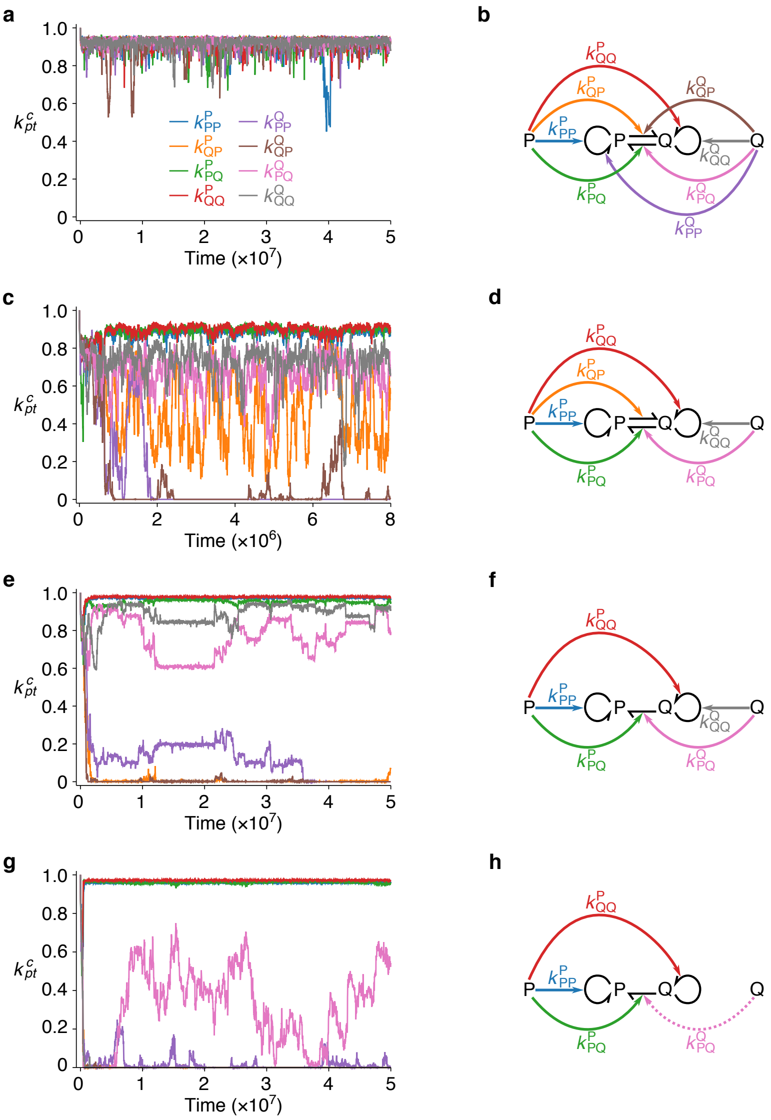

Figure S1: The evolutionary dynamics of the agent-based model. **a**, The dynamics of k_{pt}^{c} averaged over all replicators for parameters corresponding to 'no symmetry breaking' in Fig. 2a: $V = 178$ and $m = 0.01$. **b**, Catalytic activities evolved in a. **c**, **d**, Parameters corresponding to 'uncategorised' in Fig. 2a: $V = 178$ and $m = 0.1$. **e**, **f**, Parameters corresponding to 'incomplete symmetry breaking' in Fig. 2a: $V = 562$ and $m = 0.01$. **g**, **h**, Parameters corresponding to 'incomplete symmetry breaking' in Fig. 2a: $V = 1778$ and $m = 0.01$.

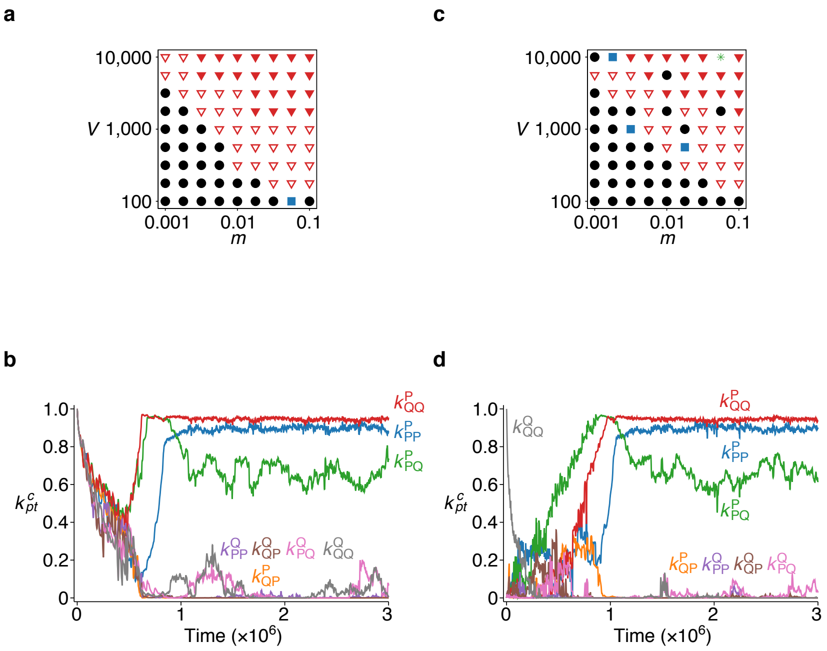

Figure S2: Symmetry breaking with an alternative definition of complex formation rates (see Supplementary Material Text 1.1). The rate constants of complex formation were defined in such a way that coexistence between P and Q is neither favoured nor disfavoured by cellular-level selection. **a**, Phase diagram with a symmetric initial condition: $k_{pt}^{c} = 1$ for all combinations of c, p, and t, with both P and Q present at the beginning of each simulation. The symbols are the same as in Fig. 2a, except that the circles include cases in which one replicator type goes extinct. **b**, Dynamics of k_{pt}^{c} averaged over all replicators for $m = 0.01$ and $V = 10000$ in a. **c**, Phase diagram with an asymmetric initial condition: $k_{\mathrm{QQ}}^{\mathrm{Q}} = 1$ and $k_{pt}^{c} = 0$ for all the other combinations of c, p, and t, with only Q present at the beginning of each simulation. The symbols are the same as in a, except that stars indicate the extinction of replicators. **d** Dynamics of k_{pt}^{c} averaged over all replicators for $m = 0.01$ and $V = 10000$ in b.

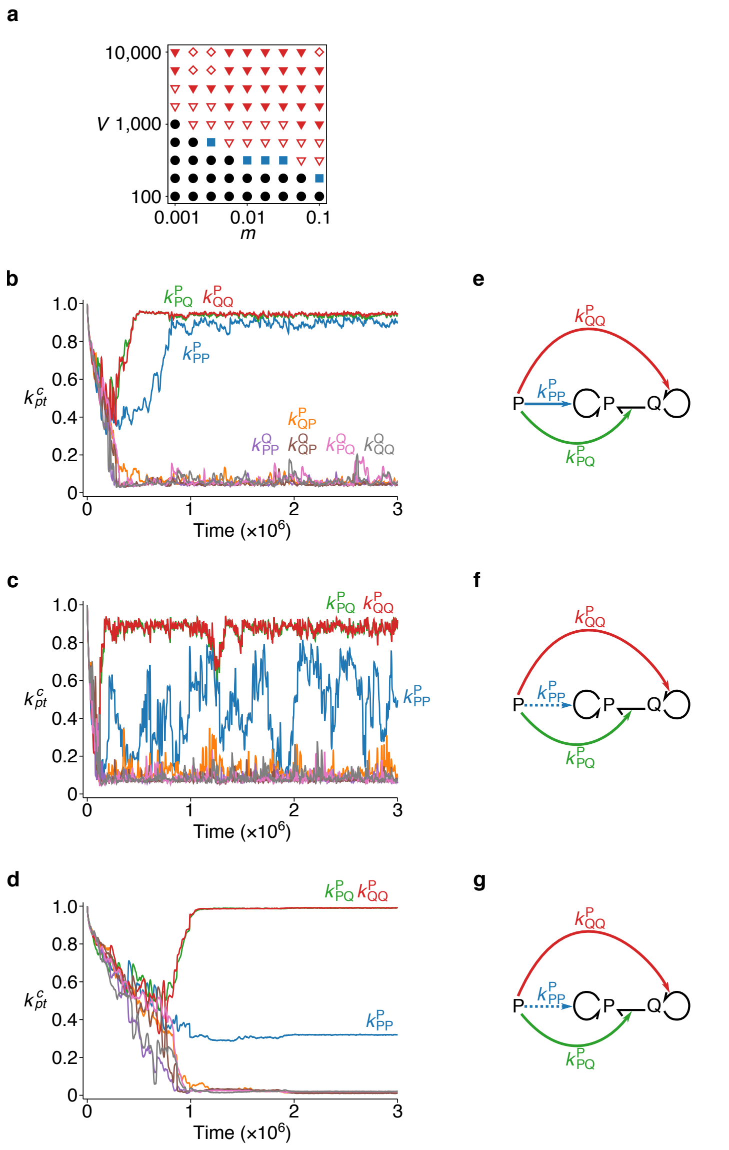

Figure S3: Symmetry breaking with reflecting mutation (see Supplementary Material Text 1.2). The mutation of k^c_{pt} is modelled as unbiased random walk with reflecting boundaries at 0 and 1. **a**, Phase diagram. The symbols are the same as in Fig. 2a ($t_{\min} > 3.9 \times 10^7$ for $m = 0.1$ and $V = 10000$). **b** Dynamics of k^c_{pt} averaged over all replicators. $m = 0.01$ and $V = 10000$. Three-fold symmetry breaking occurs. **c**, $m = 0.0562$ and $V = 10000$. Numerical symmetry breaking is slight. **d**, $m = 0.00178$ and $V = 10000$. Numerical symmetry breaking is slight. **e**, **f**, **g**, Catalytic activities evolved in b, c, d, respectively.

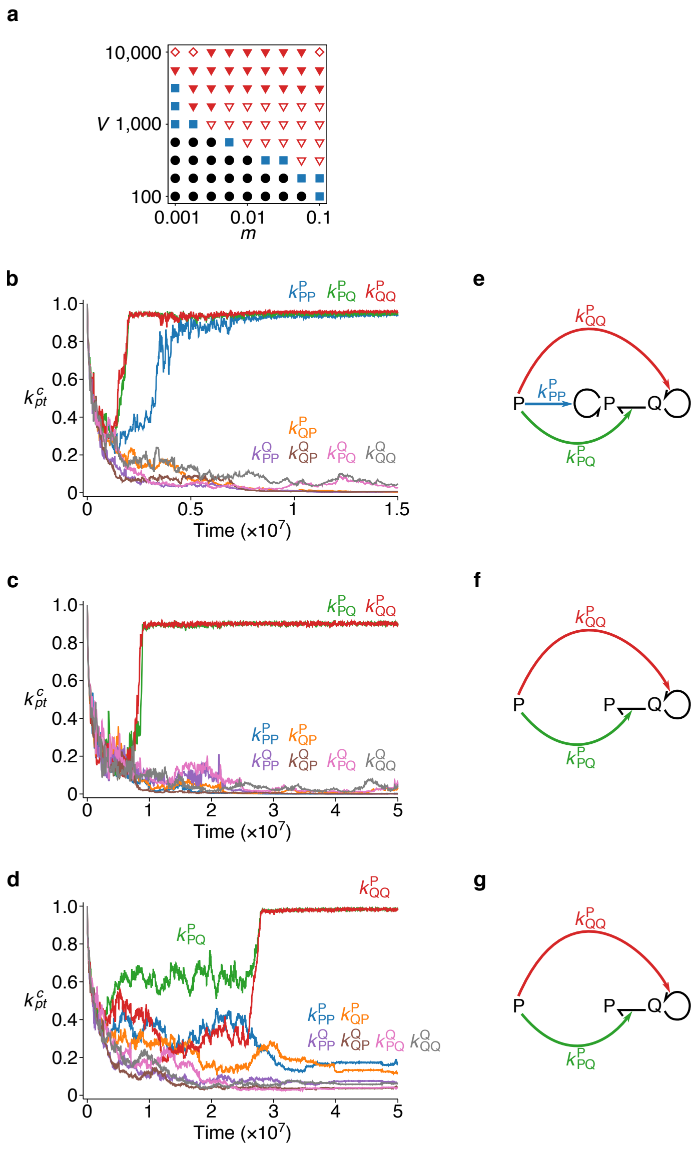

Figure S4: Symmetry breaking with log-space mutation (see Supplementary Material Text 1.2). The mutation of k^c_{pt} is modelled as unbiased random walks on a logarithmic scale. **a**, Phase diagram. The symbols are the same as in Fig. 2a ($t_{\min} > 3.9 \times 10^7$ only for $m = 0.1$ and $V = 10000$). **b**, Dynamics of k^c_{pt} averaged over all replicators. $m = 0.01$ and $V = 10000$. Three-fold symmetry breaking occurs. **c**, $m = 0.1$ and $V = 10000$. No numerical symmetry breaking occurs. **d**, $m = 0.00178$ and $V = 10000$. No numerical symmetry breaking occurs. **e**, **f**, **g**, Catalytic activities evolved in b, c, d, respectively.

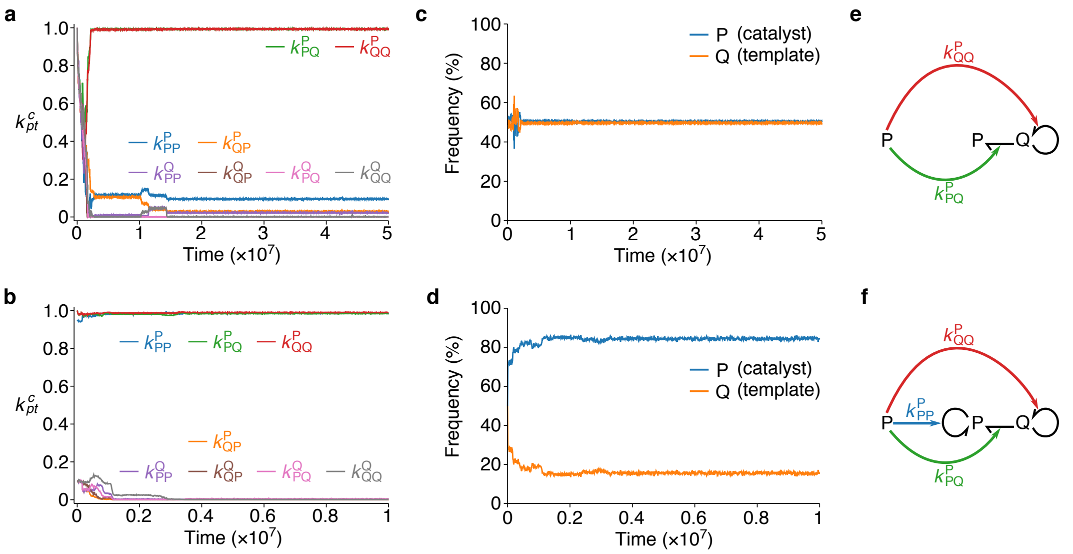

Figure S5: The absence of numerical symmetry breaking for small m and large V (see Supplementary Material Text 1.4). **a**, **b**, The dynamics of k^c_{pt} averaged over all replicators is shown for $V = 10000$ and $m = 0.001$ with two different initial conditions: a symmetric initial condition, where $k^c_{pt} = 1$ (a); an asymmetric initial condition, where $k^{\mathrm{P}}_{\mathrm{PP}} = 0.95$, $k^{\mathrm{P}}_{\mathrm{PQ}} = 0.1$, $k^{\mathrm{P}}_{\mathrm{QP}} = 1$, $k^{\mathrm{P}}_{\mathrm{QQ}} = 1$, and $k^{\mathrm{Q}}_{pt} = 0.1$ (b). The self-replication of catalysts does not evolve for the symmetric initial condition, whereas it is maintained for the asymmetric initial condition ($t_{\min} > 1.2 \times 10^7$). The dependence of the results on the initial conditions suggests the presence of bistability for $V = 10000$ and $m = 0.001$. **c**, **d**, The frequencies of P (catalysts) and Q (templates) are plotted as the functions of time. Numerical symmetry breaking does not occur for the symmetric initial condition, whereas it occurs for the asymmetric initial condition. The results indicate that numerical asymmetry depends on the self-replication of catalysts. **e**, **f**, Catalytic activities evolved for the symmetric initial condition (e) and for the asymmetric initial condition (f).

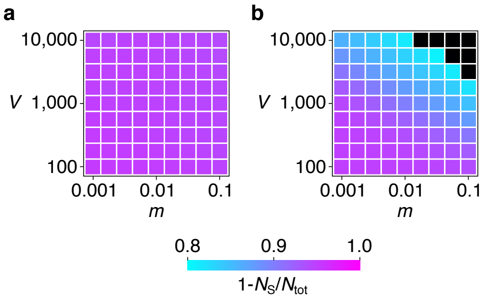

Figure S6: The effect of symmetry breaking on catalytic activities. The fraction of replicators $1 - N_{\mathrm{S}}/N_{\mathrm{tot}}$, which is a proxy for the overall catalytic activity of replicators, is shown as a function of m and V, where N_{S} is the total number of S molecules in the system, and $N_{\mathrm{tot}} = N_{\mathrm{P}} + N_{\mathrm{Q}} + N_{\mathrm{S}}$. **a**, The original model, which allows symmetry breaking (i.e., Fig. 1). **b**, The model that excludes the possibility of symmetry breaking; specifically, it allows only one type of replicator (either P or Q). Black squares indicate extinction (i.e. $N_{\mathrm{tot}} = N_{\mathrm{S}}$). $t_{\mathrm{min}} > 1.5 \times 10^7$.

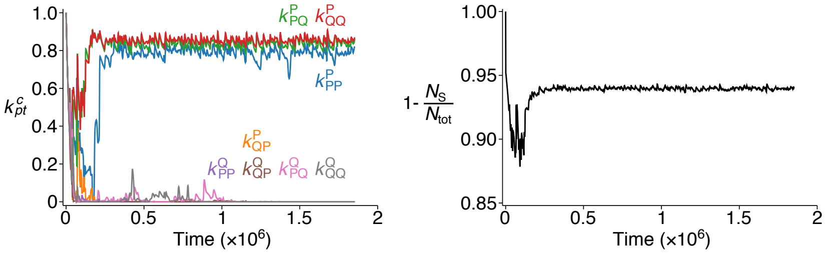

Figure S7: Result for large m and V values. The dynamics of the agent-based model is shown for $m = 0.1$ and $V = 10^5$, parameters outside the range examined in Fig. 2a and Fig. S6a. **a**, The dynamics of k^{c}_{pt} averaged over all replicators. **b**, The dynamics of the fraction of replicators $1 - N_\mathrm{S}/N_\mathrm{tot}$, where N_tot and N_S are the total numbers of particles and S molecules in the system, respectively. $t_\mathrm{min} > 1.8 \times 10^6$.

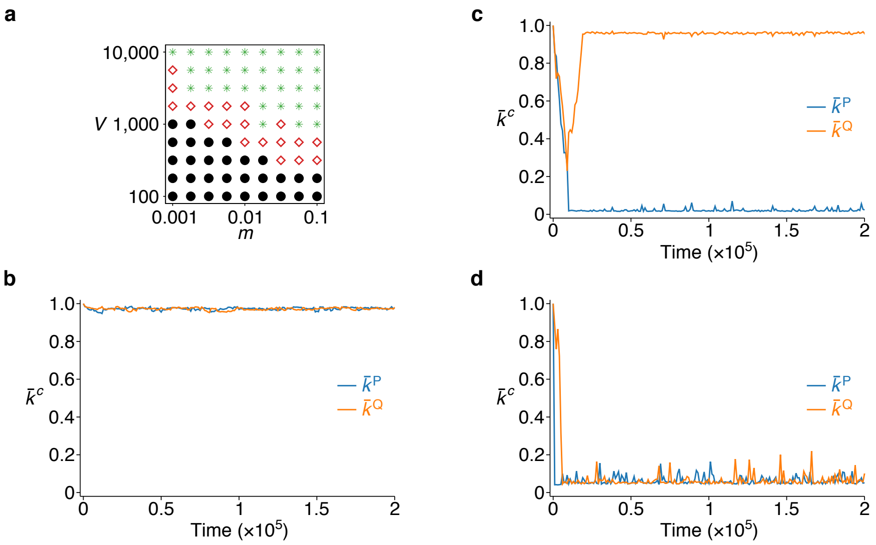

Figure S8: Symmetry breaking in a hierarchical Wright-Fisher model (see Supplementary Material Text 1.5). The model stochastically simulates the population dynamics described by equations (1), treating σ^2_{mol} and σ^2_{cel} as variables dependent on m and V (see Supplementary Material Text 1.5). **a**, Phase diagram. Circles indicate no symmetry breaking (i.e., $\bar{k}^{\text{P}} \approx \bar{k}^{\text{Q}} \approx 1$); diamonds, symmetry breaking (i.e., $\bar{k}^c \approx 0$ and $\bar{k}^{c'} \approx 1$ for $c \neq c'$); stars, extinction (i.e., $\bar{k}^{\text{P}} \approx \bar{k}^{\text{Q}} \approx 0$). $s = 1$ (cost-benefit ratio). The total number of replicators was $50V$ (approximately 130 protocells throughout simulations). The initial condition was $k^{\text{P}} = k^{\text{Q}} = 1$ for all replicators. Each simulation was run for 4×10^5 generations. The extinction (i.e., $\bar{k}^{\text{P}} \approx \bar{k}^{\text{Q}} \approx 0$) for large m and V is consistent with the phase-plane analysis of equations (1), which also shows extinction (i.e., $\bar{k}^{\text{P}} \approx \bar{k}^{\text{Q}} \approx 0$) for sufficiently large $\sigma^2_{\text{mol}}/\sigma^2_{\text{cel}}$ (parameters outside the range examined in Fig. 3). The discrepancy between Fig. S8a and Fig. 2a is due the simplifying assumption made in equations (1) that k^c_{pt} is independent of p and t. If k^c_{pt} is allowed to depend on p and t, the flow of information from templates to catalysts can become completely unidirectional. Such unidirectional flow of information can resolve the dilemma between catalysing and templating and leads to the maintenance of high catalytic activities as described in Results. **b**, The dynamics of $\bar{k}^c$ for $m = 0.001$ and $V = 1000$ (no symmetry breaking). **c**, $m = 0.01$ and $V = 1000$ (symmetry breaking). **d**, $m = 0.1$ and $V = 1000$ (extinction).

References

[1] Price GR. Extension of covariance selection mathematics. Annals of Human Genetics. 1972;35(4):485–490.

[2] Hamilton WD. Innate social aptitudes of man: an approach from evolutionary genetics. In: Fox R, editor. Biosocial Anthoroplogy. London: Malaby Press; 1975. p. 133–153.

[3] Iwasa Y, Pomiankowski A, Nee S. The evolution of costly mate preferences II. the 'handicap' principle. Evolution. 1991;45(6):1431–1442.

[4] Kaneko K, Yomo T. On a kinetic origin of heredity: minority control in a replicating system with mutually catalytic molecules. Journal of Theoretical Biology. 2002;214(4):563–576.

[5] Takeuchi N, Hogeweg P, Kaneko K. The origin of a primordial genome through spontaneous symmetry breaking. Nature Communications. 2017;8(1):250.

[6] Maynard Smith J, Szathmáry E. The Major Transitions in Evolution. Oxford: W. H. Freeman/Spektrum; 1995.

[7] Michod RE. Darwinian Dynamics: Evolutionary Transitions in Fitness and Individuality. Princeton, NJ: Princeton University Press; 1999.

[8] Bourke AFG. Principles of Social Evolution. Oxford, UK: Oxford University Press; 2011.

[9] Rice SH. Evolutionary Theory: Mathematical and Conceptual Foundations. Sunderland, MA, USA: Sinauer Associates; 2004.

[10] Hamilton WD. Selfish and spiteful behaviour in an evolutionary model. Nature. 1970;228(5277):1218–1220.
